# Environmental Impact of a Single Combined Urine Antigen Test for Streptococcus pneumoniae and Legionella pneumophila

**DOI:** 10.1017/ash.2025.190

**Published:** 2025-09-24

**Authors:** Anshel Kenkare, Rahee Nerurkar, Rohan Goyal, Phyu Thwe, Erika Orner, Wendy Szymczak, Sarah Baron, Priya Nori

**Affiliations:** 1 Montefiore; 2 Montefiore Medical Center; 3 Montefiore Health System

## Abstract

**Background:** Healthcare produces 10% of US carbon emissions. Diagnostic stewardship can improve patient care while reducing emissions from unnecessary testing. In the Bronx, community outbreaks of Legionella pneumophila result in frequent testing, yet, most testing for Legionella and Pneumococcus are negative. The institution is transitioning from separate tests for Streptococcus pneumoniae and L. pneumophila to a single combined test. We evaluated the environmental impact of this change. **Methods:** To approximate emissions from separate versus combined testing kits, each kit component was weighed and converted to CO2 emission equivalents using the US Environmental Protection Agency (EPA) greenhouse gas equivalencies calculators. Using data from the previous 12 months and projecting similar future testing patterns, we estimated an emissions reduction based on combined testing. By multiplying waste emissions before and after combined testing, we were able to estimate the emissions diverted by this institutional change. **Results:** From January 2023 to November 2024, 13906 urine Legionella tests were performed and 62 (0.45%) were positive, while 12796 urine Pneumococcal tests were performed and 479 (3.74%) were positive. Separate testing kits generated 218.2 kg of plastic waste and 749.1 kg of paper waste. Projected plastic waste from January 2025 to November of 2026 using the combined test is 48.5 kg of paper waste and 64.2 kg of plastic waste. The total estimated emissions reduction is 4.34 Kg of CO2. **Conclusions:** Diagnostic stewardship via a combined urinary antigen test can reduce waste from unnecessary testing without altering workflow. This institutional change is projected to reduce the plastic waste equivalent to 8480 water bottles and reduce emissions equivalent to using 11 gallons of gasoline or charging 340 smartphones. Our calculations are likely an underestimate of total emissions diverted since we only estimated emissions produced by waste in the landfill and did not account for other associated emissions such as those produced by transporting the waste. While the impact is modest, diagnostic stewardship applied broadly is a step towards a goal of net zero.

